# Blimp1/Prdm1 Functions in Opposition to Irf1 to Maintain Neonatal Tolerance during Postnatal Intestinal Maturation

**DOI:** 10.1371/journal.pgen.1005375

**Published:** 2015-07-09

**Authors:** Arne W. Mould, Marc A. J. Morgan, Andrew C. Nelson, Elizabeth K. Bikoff, Elizabeth J. Robertson

**Affiliations:** Sir William Dunn School of Pathology, University of Oxford, Oxford, United Kingdom; The Walter and Eliza Hall Institute of Medical Research, AUSTRALIA

## Abstract

The neonatal intestine is a very complex and dynamic organ that must rapidly adapt and remodel in response to a barrage of environmental stimuli during the first few postnatal weeks. Recent studies demonstrate that the zinc finger transcriptional repressor Blimp1/Prdm1 plays an essential role governing postnatal reprogramming of intestinal enterocytes during this period. Functional loss results in global changes in gene expression patterns, particularly in genes associated with metabolic function. Here we engineered a knock-in allele expressing an eGFP-tagged fusion protein under control of the endogenous regulatory elements and performed genome wide ChIP-seq analysis to identify direct Blimp1 targets and further elucidate the function of Blimp1 in intestinal development. Comparison with published human and mouse datasets revealed a highly conserved core set of genes including interferon-inducible promoters. Here we show that the interferon-inducible transcriptional activator Irf1 is constitutively expressed throughout fetal and postnatal intestinal epithelium development. ChIP-seq demonstrates closely overlapping Blimp1 and Irf1 peaks at key components of the MHC class I pathway in fetal enterocytes. The onset of MHC class I expression coincides with down-regulated Blimp1 expression during the suckling to weaning transition. Collectively, these experiments strongly suggest that in addition to regulating the enterocyte metabolic switch, Blimp1 functions as a gatekeeper in opposition to Irf1 to prevent premature activation of the MHC class I pathway in villus epithelium to maintain tolerance in the neonatal intestine.

## Author Summary

The transcriptional repressor Blimp1/Prdm1 plays a pivotal role in the metabolic switch that occurs in the small intestine during the suckling to weaning transition. Notably, expression profiling of perinatal Blimp1-deficient small intestine revealed premature activation of metabolic genes normally restricted to post-weaning enterocytes. To further elucidate the function of Blimp1 in intestinal development, we engineered a novel Blimp1-eGFP-fusion knock-in mouse strain to perform ChIP-seq analysis. In addition to identifying which metabolic genes are direct Blimp1 targets, ChIP-seq analysis revealed a highly conserved Blimp1/Irf-1 overlapping sites that function to control MHC class I antigen processing during acquisition of neonatal tolerance in the first weeks after birth during early colonization of the intestinal tract by commensal microorganisms. Moreover, immunohistochemical analysis of human fetal intestine suggests that a BLIMP1/IRF-1 axis may also function in human intestinal epithelium development.

## Introduction

The zinc finger transcriptional repressor Blimp1 originally cloned as a negative regulator of beta-interferon gene expression [1] is known to control cell fate decisions in the developing embryo and adult organism [2]. Blimp1 acting as a master regulator of plasma cell differentiation directly represses expression of key transcription factor genes such as *c-Myc*, *Id3*, *CIITA* and *PAX5* required for B-lymphocyte function and proliferation [3,4]. In the early embryo, Blimp1 silences the default somatic pathway and instructs a discrete subset of epiblast cells exposed to the highest levels of BMP4 signaling at the base of the allantois to become primordial germ cells (PGC) [5,6]. At later stages, Blimp1 regulates development of the posterior forelimb, caudal pharyngeal arches, secondary heart field, and sensory vibrissae [7]. Blimp1 also plays an essential role in placental morphogenesis, governing terminal differentiation of the invasive spiral artery-associated trophoblast giant cells [8].

Considerable data strongly suggests that Blimp1 transcriptional targets are cell type-specific. For example Blimp1 directly represses *c-Myc* expression to block proliferation in B cells, macrophages, and sebaceous gland progenitors [4,9,10]. In contrast however *c-Myc* is not a key target in activated effector T cells. Rather Blimp1 blocks IL-2 production required for T cell proliferation [11,12]. Within the CD4^+^ T-cell lineage Blimp1 selectively attenuates Th1 subset development by extinguishing expression of *IFNγ*, *Tbx21* and *Bcl6* [13]. *Nfat5*, *Fos*, *Dusp16* and *Prdm1* itself are direct targets in the skin epidermis [14]. Recent ChIP-seq experiments analyzing transfected P19 embryonic carcinoma (EC) cells demonstrate that Blimp1 directly represses numerous developmental and somatic regulators [15]. Cooperative binding with AP2γ and Prdm14 dramatically shifts gene expression profiles and initiates the transcriptional programme required for PGC specification [15].

Blimp1 is strongly expressed in the intestinal epithelium throughout fetal development but beginning at birth becomes dramatically down-regulated in the crypt progenitors, corresponding to the adult intestinal stem cell compartment [7,16,17]. Conditional deletion experiments revealed an essential role in governing postnatal reprogramming of intestinal enterocytes during the suckling to weaning transition [16,18]. Transcriptional profiling experiments demonstrate Blimp1 functional loss results in global changes in gene expression patterns. Thus numerous immature enterocyte markers including digestive enzymes required for processing maternal milk were markedly reduced, whereas in contrast several key components of the adult biochemical signature were substantially and prematurely activated [16].

To further investigate Blimp1 functional contributions during this developmental transition and identify its direct targets in immature intestinal enterocytes, we created a knock-in allele engineered to express an enhanced green fluorescent protein (eGFP)-tagged fusion protein reactive with the well-characterized ChIP quality anti-GFP monoclonal antibody. Here we demonstrate that the fusion protein faithfully reconstitutes Blimp1-dependent functional activities. Thus homozygous embryos exclusively expressing the eGFP-tagged Blimp1-fusion protein develop normally, and healthy homozygous adults recovered at the predicted Mendelian ratios were indistinguishable from wild type littermates. Moreover, the knock-in allele efficiently rescues Blimp1-dependent plasma cell differentiation. EGFP-tagged Blimp1 was strongly expressed in the developing intestine allowing us to undertake unbiased ChIP-seq analysis. Several candidate target genes strongly up-regulated in conditional loss mutants were identified as direct targets including *Cyp4v3*, *Slc16a5* and *Myo18b*. Interestingly, comparisons of our ChIP-seq peaks with those reported for P19 EC cell and human HeLa cell datasets revealed several highly conserved genomic targets, including key components of the MHC class I peptide-loading pathway [19].

SELEX experiments revealed that the Blimp1 consensus-binding motif closely resembles the IRF-E sequence [20] recognized by IRF1, an activator of β-interferon gene expression [21–23]. Recent studies suggest that competitive BLIMP1 and IRF1 binding regulates expression of IFN-inducible components of the MHC class I peptide loading machinery [24]. Here we performed genome-wide ChIP-seq analysis to define the extent of overlap between Blimp1 and Irf1 occupancy *in vivo* under physiological conditions in the intestinal epithelium. We found overlapping Blimp1/Irf1 binding sites proximal to the promoters of 24% (24 of 99) of human/mouse conserved Blimp1 target genes including *Psmb8*, *Psmb10*, *Psme1*, *Tapbp* and *Erap1*. These findings strengthen the idea that Blimp1 occupancy directly antagonizes Irf1-dependent activation of MHC class I antigen presentation. Consistent with this suggestion we demonstrate that Irf1 is constitutively expressed in the fetal intestine and throughout postnatal stages. The onset of MHC class I expression in the developing intestine precisely coincides with down-regulated Blimp1 expression and the appearance of crypt-derived Blimp1-negative adult enterocytes during the suckling to weaning transition. Besides its role in governing the switch to adult metabolic pathways, Blimp1 co-occupancy at these Irf1-target genes promotes neonatal tolerance in the first weeks after birth during early colonization of the intestinal tract by commensal microorganisms.

## Results

### The eGFP-tagged Blimp1 knock-in allele rescues plasma cell differentiation and is efficiently expressed in the developing placenta and embryonic small intestine

**Fig 1:**
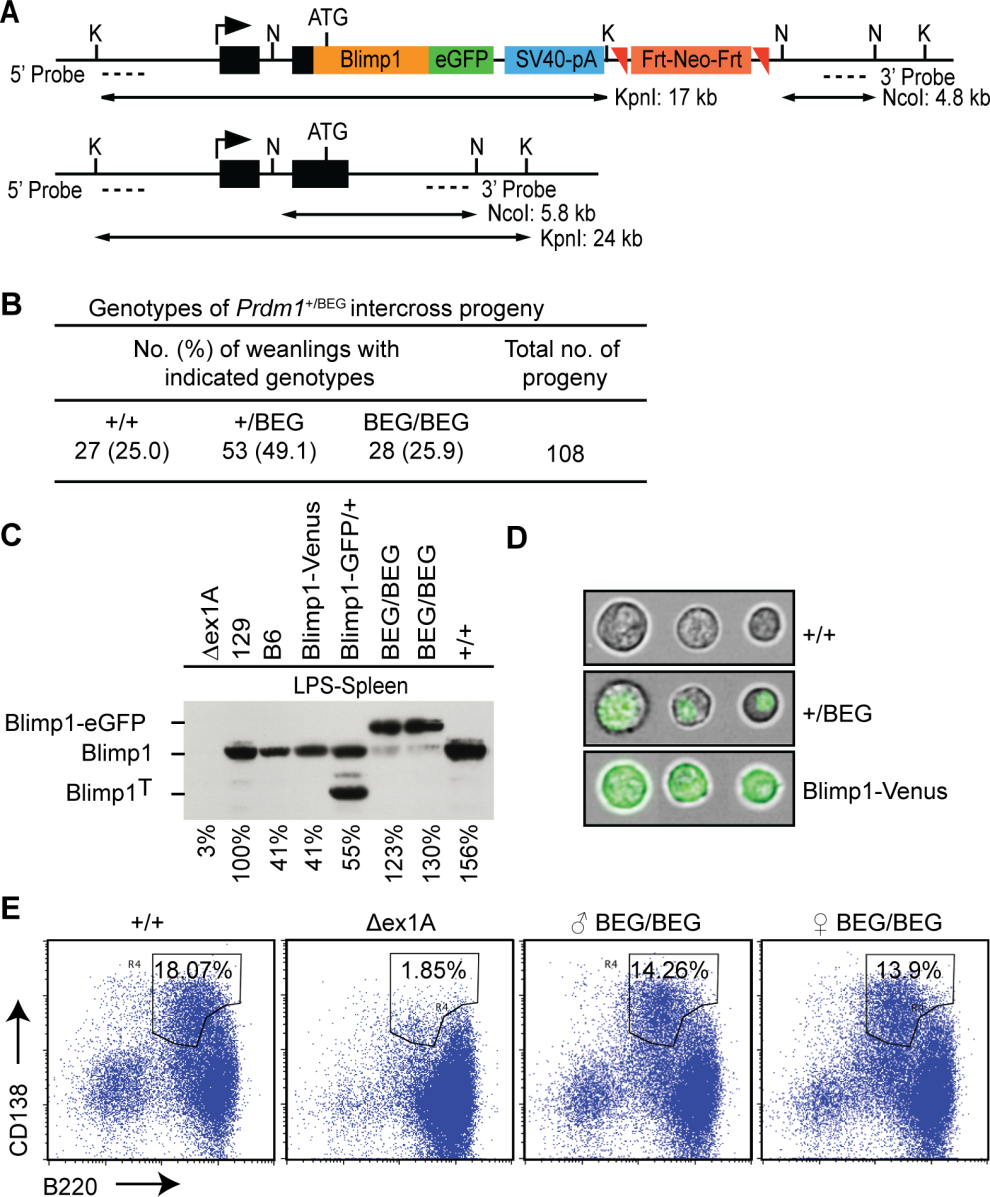
The eGFP-tagged Blimp1 knock-in allele efficiently rescues plasma cell differentiation. (A) Schematic representation of the targeting vector, wild type locus, and Southern blot screening strategy. (B) Intercross matings of heterozygous BEG animals generate Mendelian ratios of wild type, heterozygous and homozygous mutant progeny. (C) Western blot analysis demonstrates the eGFP-Blimp1 fusion protein is robustly induced in LPS-treated splenocytes. Levels of Blimp1 protein were quantified relative to the 129/SvEv wild type sample. Additional positive controls were wild type C57BL/6 and a BEG intercross littermate (+/+), heterozygous Blimp1-Venus BAC transgene (Blimp1-Venus) [25] or Blimp1-IRES-GFP (Blimp1-GFP/+) [26] reporter strains. Prdm1ΔEx1A splenocytes lacking the ability to generate plasma cells were included as a negative control [27]. (D) Imagestream analysis of LPS-treated splenocytes reveals nuclear localization of the Blimp1-eGFP fusion protein in contrast to the cytoplasmic/plasma membrane localization of Venus expressed under the control of the Blimp1 BAC-transgene. (E) Homozygous mutant B cells that exclusively express the eGFP-tagged Blimp1-fusion protein efficiently undergo plasma cell (CD138+B220+) terminal differentiation.

To enable identification of Blimp1 target genes in diverse embryonic and adult tissues *in vivo* under physiological conditions, we engineered a novel eGFP-tagged Blimp1 knock-in allele *Prdm1* ^BEG^ by introducing the cDNA expression cassette into the first coding exon (Fig 1A). This strategy, used successfully for construction of a *Prdm1 cre LacZ* reporter allele, preserves all known regulatory and structural features of the endogenous locus [8]. Consistent with this homozygous embryos exclusively expressing the Blimp1-eGFP fusion protein develop normally. Additionally, healthy weanlings recovered from intercross matings at the predicted Mendelian ratios, were indistinguishable from wild type littermates (Fig 1B) and display no signs of disease when housed in a specific pathogen free environment.

**Fig 2:**
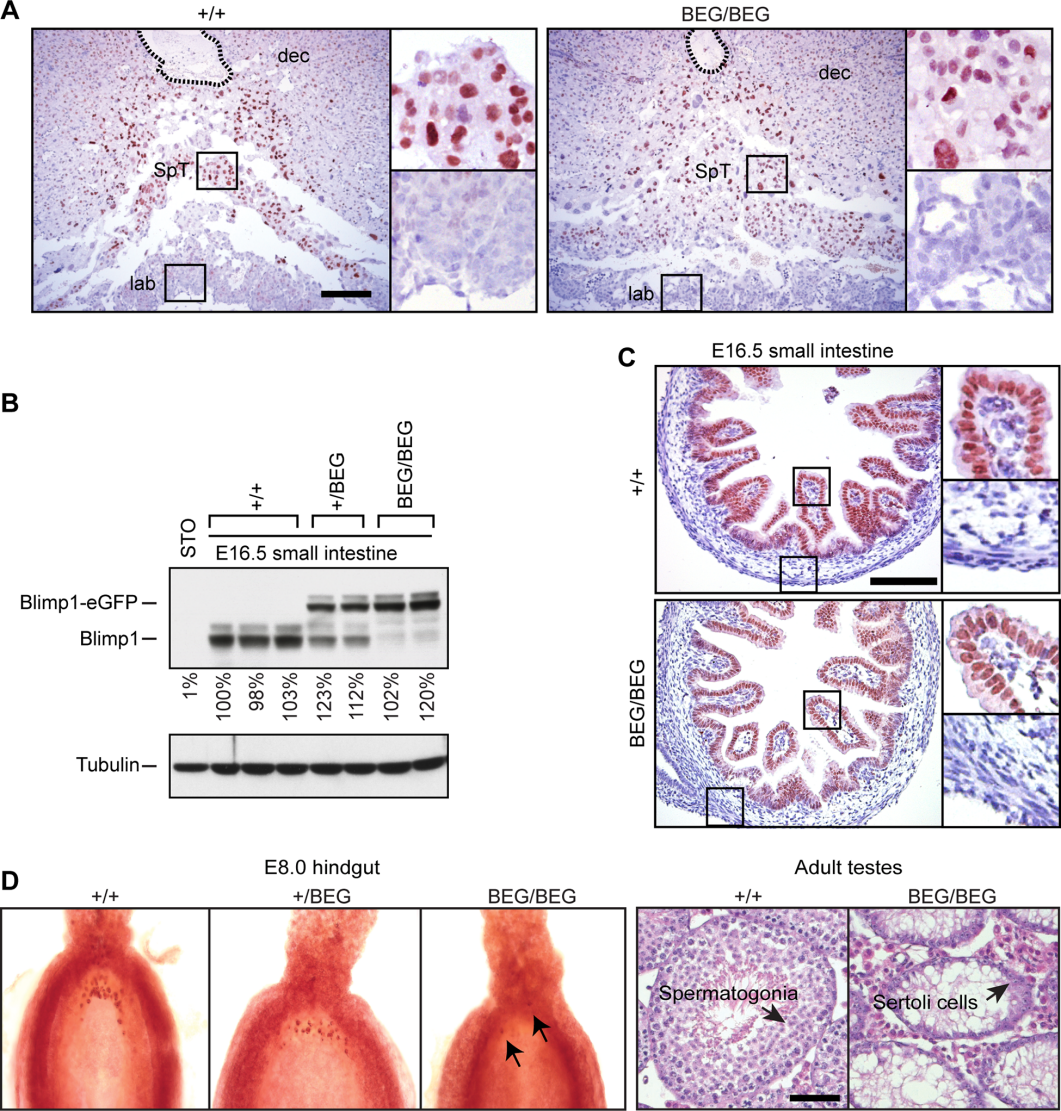
The eGFP-tagged Blimp1-fusion protein is efficiently expressed in the developing placenta and embryonic small intestine, but fails to rescue germ cell defects. (A) Immunohistochemical staining of E9.5 placentae demonstrates the eGFP-tagged Blimp1-fusion protein is correctly expressed in the spongiotrophoblast layer. The position of the central maternal artery is outlined. Bar, 200 μm. SpT, spongiotrophoblast; dec, maternal decidua; lab, labyrinth trophoblast. (B) Western blot analysis demonstrates robust expression in E16.5 small intestine. RIPA lysates of STO fibroblasts are included as a negative control. Levels of Blimp1 expression were quantified relative to the first wild type littermate. (C) Immunohistochemical staining demonstrates nuclear expression of both endogenous and eGFP-tagged Blimp1 protein in E16.5 villus epithelium. Bar, 200 μm. (D) The eGFP-tagged Blimp1 knock-in allele fails to reconstitute germ cell formation. Fast red alkaline phosphatase staining at E7.5 demonstrates markedly reduced numbers of PGCs (black arrows). Hematoxylin- and eosin-stained sections confirm that adult homozygous BEG/BEG testes lack spermatocytes. Bar, 200 μm.

Western blot experiments demonstrate the eGFP-tagged fusion protein is strongly expressed in LPS-stimulated splenocytes (Fig 1C). The eGFP-fusion protein efficiently translocates to the nucleus (Fig 1D) and rescues plasma cell differentiation (Fig 1E). Robust expression in the spongiotrophoblast and invading spiral artery trophoblast cells in E9.5 *Prdm1* ^BEG/BEG^ placenta faithfully reconstitutes Blimp1 functional requirements (Fig 2A)[8]. Western blot analysis demonstrates robust expression in the embryonic small intestine (Fig 2B). Immunohistochemical staining confirmed intestinal expression is restricted to the villus epithelium (Fig 2C). Small intestine tissue architecture and body weights were indistinguishable from wild type littermates at all stages examined.

Strikingly however, in contrast to mice carrying the exon 1A deletion with modestly reduced expression levels [27], here we found that healthy adult homozygous animals were sterile. Furthermore, as judged by fast red alkaline phosphatase staining, embryos exclusively expressing the knock-in allele have greatly diminished numbers of PGCs and adult testes lack spermatocytes (Fig 2D). These results demonstrate that the knock-in allele lacks the ability to induce the germ cell transcriptional programme. It is of course possible that functionality of the eGFP-tagged fusion protein is selectively compromised in PGCs due to its inability to recruit the entire cohort of epigenetic partners necessary for silencing of the default somatic pathway. On the other hand, Blimp1 functional requirements in the germ cell lineage are known to be exquisitely dose-dependent [5,6]. In all likelihood the failure to rescue germ cell defects simply reflects inadequate expression levels.

**Fig 3:**
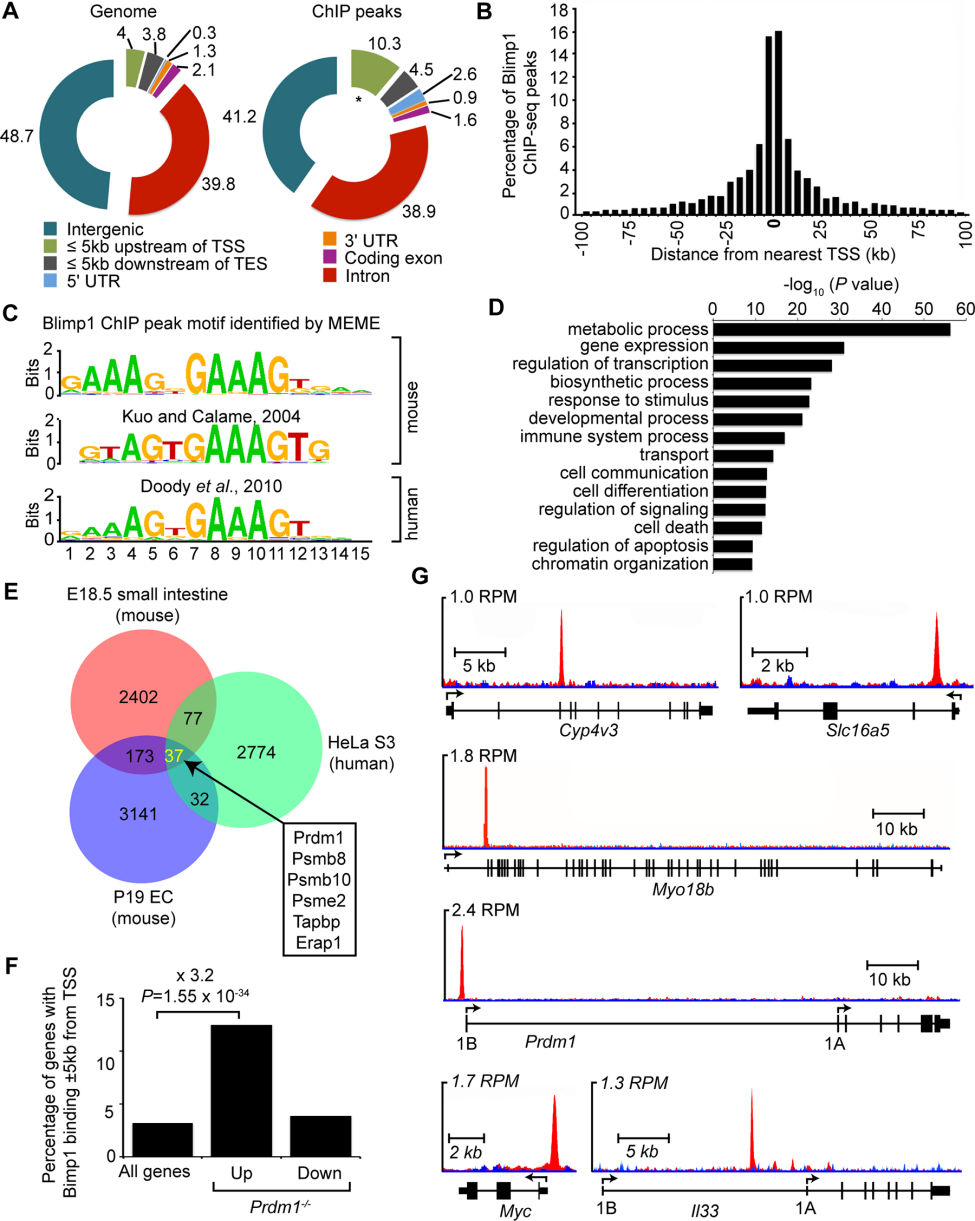
ChIP-seq analysis of genome-wide Blimp1 binding sites in E18.5 small intestine. (A) Distribution of all Blimp1 peak locations in comparison to whole genome at defined genomic regions. * *P* = 2.5 x 10^−45^ in comparison to whole genome for the same region. (B) The distance of each Blimp1 ChIP-seq peak from the nearest TSS binned at 5 kb intervals. (C) *De novo* motif analysis (MEME) identifies significant enrichment of a consensus Blimp1 binding motif underlying Blimp1 peaks (n = 2689, MEME E value = 4.7 x 10^−712^) that closely resembles previously reported mouse (STAMP E value = 3.4 x 10^−7^) and human BLIMP1 motifs (TOMTOM *P* value = 2.8 x 10^−9^). (D) Functional annotation analysis using GREAT reveals Blimp1 preferentially binds to promoter regions upstream of genes associated with metabolism, transcription, developmental and immune processes. (E) A subset of overlapping Blimp1 peaks shared with previously reported mouse and human ChIP-seq datasets. (F) Up-regulated genes in conditional mutants are significantly (*P* = 1.1 x 10^−22^) enriched for Blimp1 binding (+/- 5 kb of TSS) relative to all genes on the array. (G) UCSC track view of ChIP (red) and input (blue) wiggle plot overlays showing enrichment of GFP ChIP-seq density in *Prdm1* ^BEG/BEG^ embryonic small intestine proximal to the promoters of *Cyp4v3*, *Slc16a5*, *Myo18b*, *Prdm1*, *Myc* and *Il33*.

To identify Blimp1 targets in the developing intestine a well-characterized anti-GFP mouse monoclonal antibody [15,28] was exploited for ChIP-seq analysis. We identified 2689 Blimp1 binding events in embryonic (E18.5) small intestine (S1 Dataset). Blimp1 binding proximal to selected target genes was validated by ChIP-qPCR (S1 Fig). Analysis of peak locations relative to gene annotations revealed a broad distribution of Blimp1 binding throughout the genome with a bias towards regions proximal to TSSs (Fig 3A and 3B). A comparable number of ChIP-seq peaks (n = 3018) were recently documented in transfected P19 embryonic carcinoma cells over-expressing the identical eGFP-tagged Blimp1 fusion protein [15]. *De novo* motif analysis of sequences underlying all 2689 Blimp1 peaks revealed a highly significant consensus DNA binding motif (Fig 3C) that closely resembles the canonical Blimp1 binding motif originally identified in SELEX experiments [20], as well as that identified via ChIP-on-Chip analysis of a human myeloma cell line [29].

### Blimp1 occupies the proximal promoters of a subset of highly conserved core genomic targets

Functional annotation of genes with proximal Blimp1 binding revealed significant enrichment of GO terms associated with metabolic process and transcriptional regulation (Fig 3D). Comparisons with ChIP-seq peaks recently identified in transfected embryonic carcinoma cells [15] revealed a small subset of overlapping peaks corresponding to roughly 7–8% of each dataset (Fig 3E). The majority (171 out of 210) fall within 5 kb of annotated gene TSSs. Additionally, comparisons with peaks identified in human HeLa S3 cells [30] revealed 114 highly conserved core Blimp1 genomic targets, including 35 genes (37 peaks) universally present within all three datasets (Fig 3E and S2 Dataset). Interestingly, IFN-inducibility is a common feature shared by several of these components of the MHC class 1 antigen processing machinery i.e. Psmb8, Psmb10, Psme2, Tapbp and Erap1 [19,31].

Numerous up-regulated genes previous identified in our transcriptional profiling experiments contain predicted Blimp1 binding sites proximal to their promoter regions [16]. To evaluate whether these candidates are direct Blimp1 target genes, we compared the list of significantly altered genes (*P*<0.05 with Benjamini and Hochberg multiple testing correction) with the present ChIP-seq Blimp1 binding events. We observed significant enrichment of Blimp1 binding sites proximal to up-regulated genes (Fig 3F). In contrast, there was no significant overlap between genes with proximal Blimp1 binding and those having decreased expression in conditional mutants. Genes with markedly increased expression that show proximal Blimp1 binding (S3 Dataset) include *Cyp4v2*, *Slc15a5*, *Myo18b* and *Il33* (increased 27.7, 16.1 16.5 and 4.6 fold in mutants, respectively, Fig 3G). Notably, as judged by these criteria, *Il33* expressed at high levels in epithelial barrier tissues and believed to play a key role in amplifying innate immunity [32,33], is a direct Blimp1 target. Surprisingly Sucrase isomaltase (*Sis*) (365.2 fold increased in mutants) predicted *in silico* to contain multiple Blimp1 binding sites proximal to its promoter [16] lacks occupancy. Similarly, Arginase type 2 (*Arg2*) (174.5 fold increased in mutants) gave no detectable ChIP-seq peak. *In vivo* in the context of the E18.5 embryonic intestine, compacted chromatin near genomic regions surrounding these potential target sites appears to be inaccessible. We observed robust Blimp1 binding proximal to the *c-Myc* P1 promoter (Fig 3G). However, as previously reported expression is not significantly altered in *Prdm1* ^-/-^ small intestine [16,18]. Indeed, the vast majority of binding events detectable in the present ChIP-seq experiments play no obvious role in Blimp1-dependent transcriptional regulation, and rather seem to reflect so-called neutral occupancy [34].

Blimp1 transcripts are significantly up-regulated in conditional null *Prdm1* ^-/-^ small intestine [16]. Similarly in mouse keratinocytes an intronic Blimp1 binding site downstream of exon 3 mediates a negative feedback loop [14]. However here we failed to detect occupancy at the intronic site described by Magnusdottir *et al*. [14]. Rather we observe robust binding at the alternative distal 1B promoter region (Fig 3G)[27] that drives expression in the yolk sac endoderm [27] and embryonic intestine (S2 Fig). This highly conserved ChIP-seq peak was universally present in both mouse and human datasets [15,30]. Interestingly the sea urchin Blimp1 orthologue efficiently binds to the conserved Blimp1 consensus motif [24] and a negative autoregulatory loop upstream of its alternative promoter has been implicated in the specification of endomesodermal territories [35]. Collectively these observations suggest that Blimp1 itself is a direct target of repression but mechanistic details may be cell-type specific.

### Irf1 is constitutively expressed in the developing small intestine

**Fig 4:**
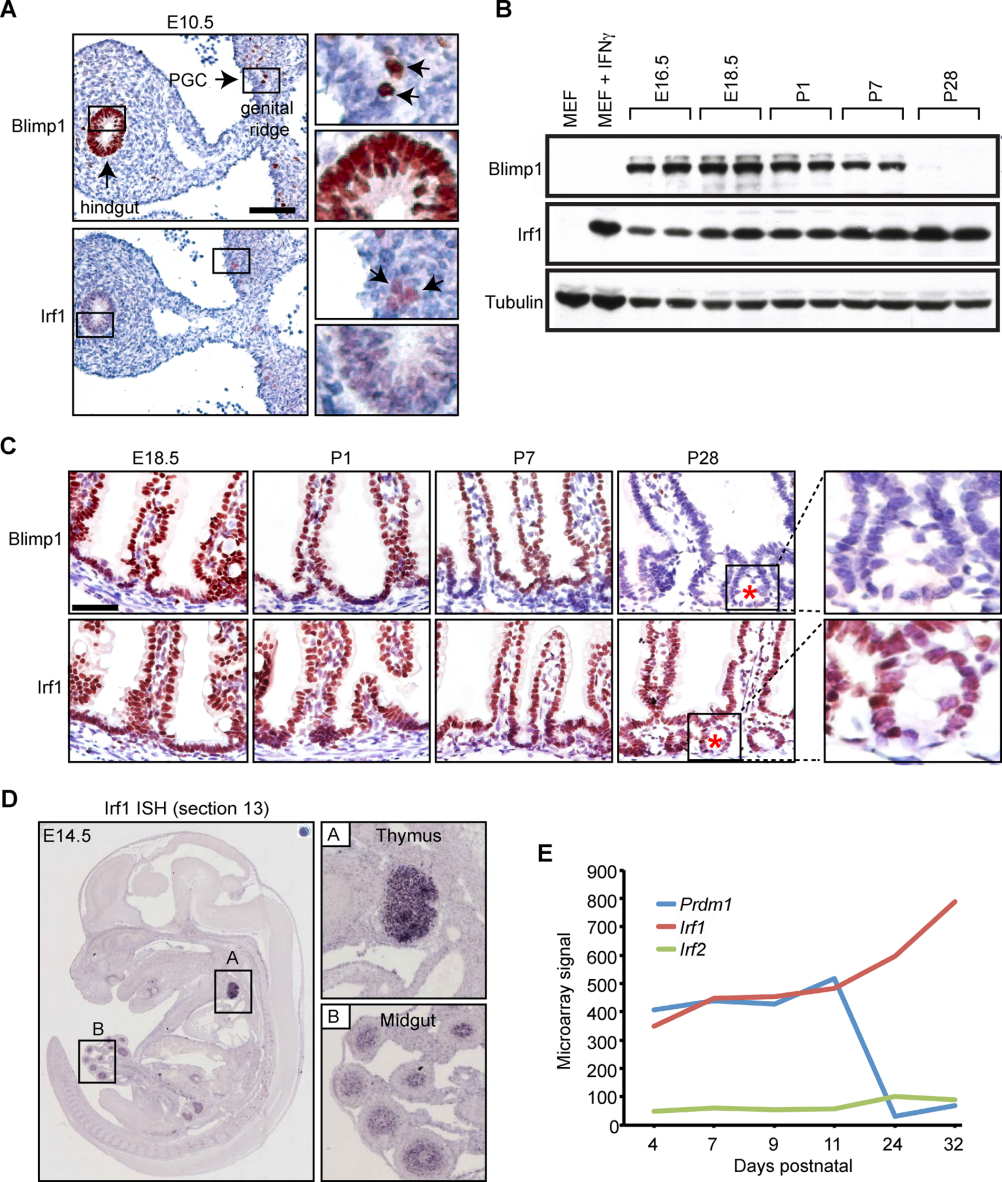
Irf1 is constitutively expressed in the developing thymus and small intestine. (A) Immunohistochemical staining reveals weak Irf1 expression in the pseudo-stratified epithelium of the developing gut tube and PGCs (black arrows) closely overlaps with robust Blimp1 expression. Bar, 100 μm. (B) Western blot analysis demonstrates Irf1 constitutively expressed in E16.5 intestine and throughout post-natal stages. In contrast, Blimp1 expression becomes down-regulated during the suckling to weaning transition. (C) Irf1 and Blimp1 nuclear staining in the villus epithelium from E18.5 to early postnatal stages (P7). Irf1 expression maintained in villus epithelium at post-weaning stages is readily detectable in the developing crypts of Lieberkuhn (indicated by red asterisk). Bar, 50 μm. (D) ISH analysis reveals *Irf1* robustly expressed in the developing thymus and midgut at E14.5 (data from EurExpress) [38]. (E) Microarray expression data (GEO accession number GDS2989) reveals abundant levels of *Irf1*, but not *Irf2* transcripts in the early postnatal small intestine are up-regulated at later stages when Blimp1 expression becomes extinguished.

It has been known for many years that the Blimp1 consensus binding motif shares a high degree of sequence overlap with IRF-E core sequence containing GAAAGT/C or GAAACT/C [20,36]. We also observed here that the IRF1 DNA binding motif was the second most significant match (*P* = 2.8 x 10^−8^) after Blimp1 to the *de novo* identified consensus DNA binding motif underlying all Blimp1 ChIP-seq peaks (S3 Fig). Irf1 expression has been previously documented in purified PGCs [37]. Similarly here, as shown in Fig 4A, we observe weak Irf1 staining in migrating PGCs and the pseudo-stratified epithelium of the primitive gut tube as early as E10.5. Western blot analysis demonstrates Irf1 abundantly expressed in the developing intestine at E16.5 (Fig 4B) is maintained during the time frame when Blimp1 becomes down regulated between postnatal day 7 and 28 [16]. Strong nuclear staining is readily detectable in both the villus epithelium and the crypts at postnatal stages (Fig 4C). Data from the online EurExpress RNA *in situ* hybridization (ISH) database [38] indicates that *Irf1* transcripts are present in the developing gut tube as early as E14.5 prior to villus formation (Fig 4D). *Irf1* is also constitutively expressed in the developing thymus. Transcriptional profiling data (Small intestine postnatal development, GEO accession number GDS2989) [39] demonstrates high levels of *Irf1* and *Prdm1*, but importantly the closely related family member *Irf2*, known to bind the same IRF-E consensus sequence as Irf1, is only minimally co-expressed in the intestine (Fig 4E)[20,36,40,41].

### Binding of Irf1 and Blimp1 overlaps at the proximal promoters of antigen processing genes

**Fig 5:**
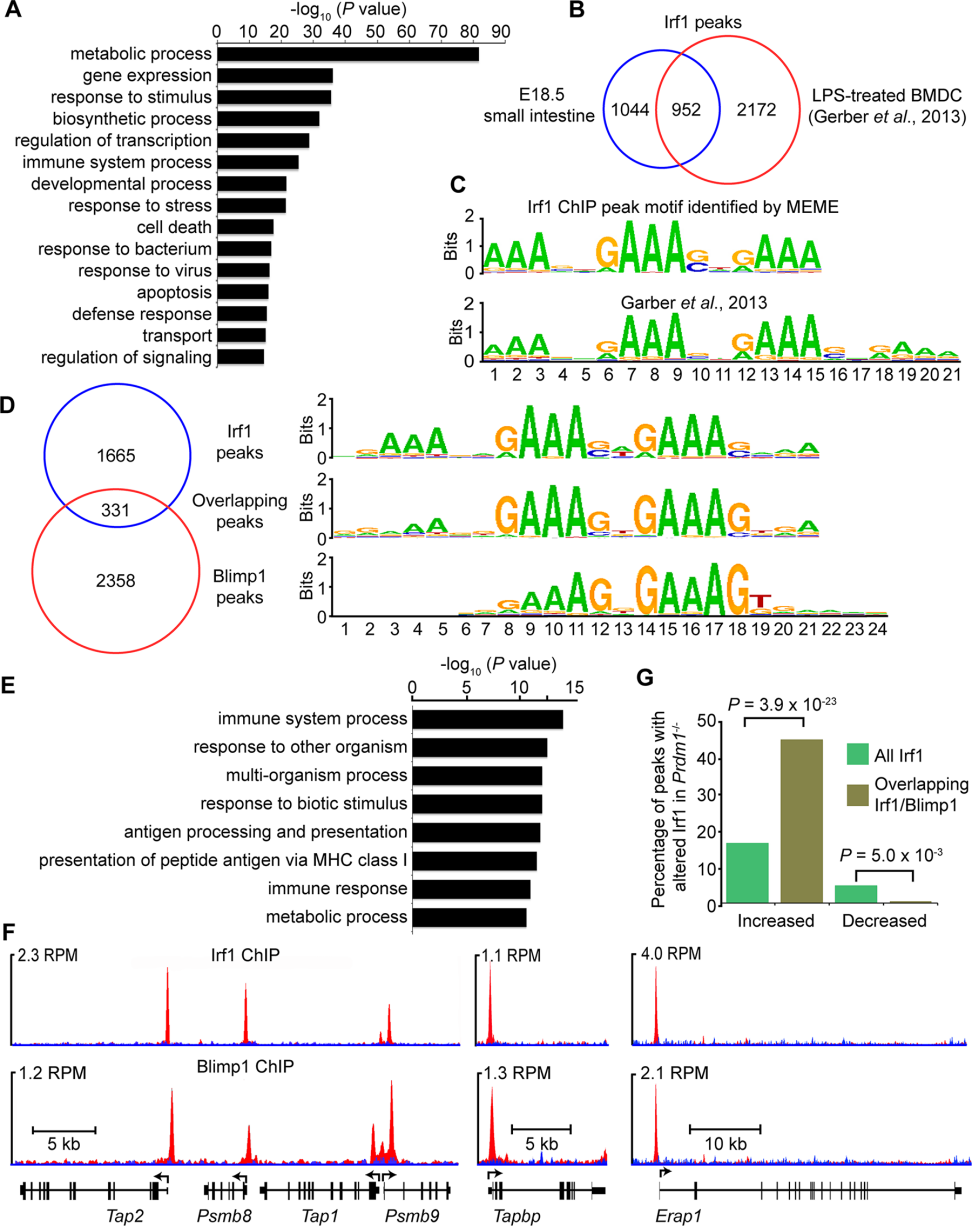
ChIP-seq analysis of genome-wide Irf1 binding sites in E18.5 small intestine. (A) Functional annotation analysis of genes bound by Irf1. (B) Overlapping Irf1 peaks between those identified in the present study and a mouse LPS-BMDC ChIP-seq dataset. (C) *De novo* motif analysis identifies significant enrichment of the consensus IRF1 binding motif underlying Irf1 peaks (n = 1996, MEME *E* value 1.9 x 10^−802^) that closely resembles the motif identified by Garber *et al*. [42] (STAMP *E* value = 0). (D) Comparison of our Irf1 and Blimp1 ChIP-seq datasets identifies a subset of overlapping peaks that selectively display the IRF1 motif. (E) Functional annotation analysis of genes bound by both Irf1 and Blimp1 demonstrates enrichment of immune response and antigen processing genes. (F) UCSC track view of ChIP (red) and input (blue) Wiggle plots. Both Irf1 and Blimp1 ChIP-seq peaks were present proximal to the promoters of *Psmb8*, *Psmb9*, *Tap1*, *Tap2*, *Tapbp* and *Erap1*. Positions of the TSS and direction of transcription are indicated by the arrows. (G) Overlapping Irf1 and Blimp1 peaks near a subset of Irf1 target genes display increased Irf1 occupancy in the absence of Blimp1 competition.

Next to directly identify Irf1 targets in the E18.5 small intestine we performed ChIP-seq analysis. We identified 1996 binding events (S4 Dataset) broadly distributed throughout the genome displaying a bias towards regions proximal to TSSs (S4A and S4B Fig) [42]. Functional annotation revealed significant enrichment for GO terms associated with immune function (Fig 5A) and antigen recognition (Fig 5E). Comparison of our Irf1 ChIP seq peak dataset with genomic coordinates previously reported for mouse LPS-treated BMDC [42] revealed a high degree of conservation (Fig 5B). Nearly half (48%) of our Irf1 peaks display a corresponding peak in the previously reported dataset (31% overlap). The *de novo* Irf1 DNA binding motif identified here and that described by Garber *et al*. [42] were indistinguishable (Fig 5C) and closely matches those described for IRF1 in primary human monocytes (*P* = 3.3 x 10^−11^) [43], Blimp1 (*P* = 3.4 x 10^−6^) as well as IRF2 (*P* = 5.1 x 10^−6^). Roughly 33% IRF1/BLIMP1 overlap was predicted in human myeloma cells [29], whereas here Irf1 and Blimp1 ChIP-seq peaks show approximately 12% (n = 331, S5 Dataset) overlap.

Functional annotation of the 331 overlapping Irf1/Blimp1 ChIP-seq peaks using GREAT revealed significant enrichment of GO terms associated with MHC class I antigen processing (Fig 5E). To evaluate competitive Blimp1 and Irf1 binding we compared the number of reads underlying Irf1 peaks in Blimp1 mutant versus wild type small intestines. Mutants displayed a significant preference for increased Irf1 occupancy at overlapping Blimp1/Irf1 sites (Fig 5G and S5 Dataset). Conversely, overlapping regions were significantly less likely to show reduced Irf1 occupancy in Blimp1 mutants.

### The onset of MHC class I expression coincides with down-regulated Blimp1 expression in the developing intestine

**Fig 6:**
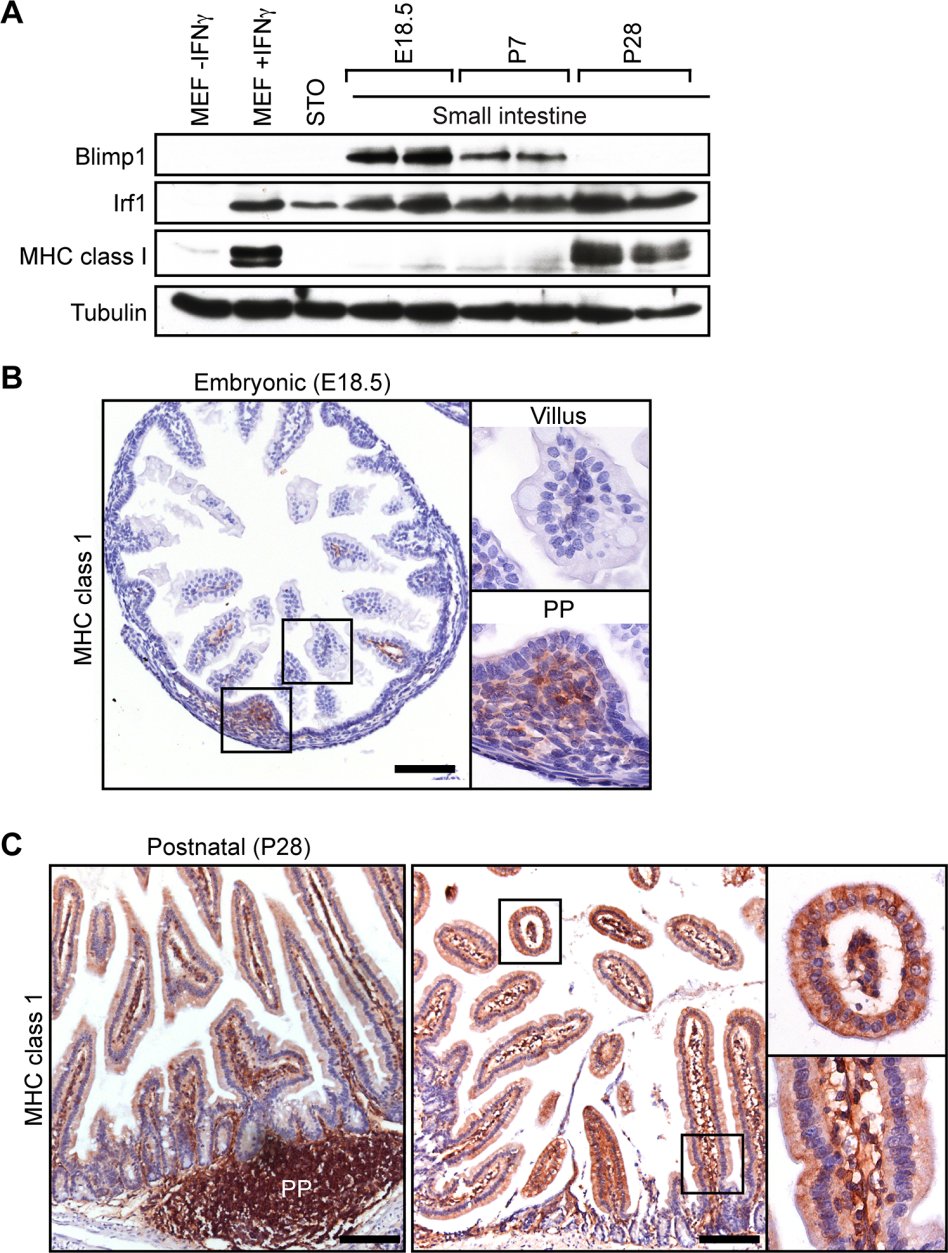
The onset of MHC class I expression coincides with down-regulated Blimp1 expression during the suckling to weaning transition. (A) Western Blot analysis reveals down-regulated Blimp1 and slightly increased Irf1 co-incident with the onset MHC class I expression detectable at post weaning stages. As a positive control Irf1 and MHC class I were strongly induced in IFNγ-treated MEFs. (B) Immunohistochemical analysis demonstrates MHC class I expression initially appears in immature Peyer’s patches (PP). Bar, 200 μm. (C) MHC class I is robustly expressed in post-natal villus epithelium (P28), as well as mature Peyer’s patches and villus mesenchyme. Bar, 200 μm.

To directly examine MHC class I expression in the developing small intestine, we performed Western blot experiments. Irf1 is strongly expressed at E16.5 and throughout postnatal intestinal development (Fig 6A). In contrast Blimp1 expression is completely lost by P28, co-incident with the onset of MHC class I expression. Immunohistochemistry similarly demonstrate MHC class I expression, readily detectable in embryonic Peyer’s patches at E18.5, increases dramatically by P28 (Fig 6B and 6C). These findings strongly suggest that Blimp1 repression of IFN-dependent components of the MHC class I peptide-loading pathway plays a key role in restraining premature activation of host immune responses *in utero* and early postnatal stages.

The ability of Blimp1 to mediate gene silencing and re-organize the chromatin landscape at its target genes depends on recruitment of histone-modifying enzymes [2]. Recent work suggests that postnatal maturation of the intestinal epithelium during the suckling to weaning transition is accompanied by global changes in the epigenetic machinery [44]. Decreased Hdac1 and Hdac2 expression was associated with a modest reduction in histone acetylation. However, protein stability may be compromised due to increased levels of digestive enzymatic activities during intestinal maturation. In snap frozen samples directly lysed in SDS sample buffer we observed consistently high levels of Blimp1 epigenetic partners including Lsd-1, G9a, Hdac1 and Hdac2 throughout the postnatal period (S5 Fig). Thus dramatic changes in transcriptional profiles during postnatal maturation of the intestinal epithelium cannot simply be explained due to developmentally regulated shifts in the composition of co-repressor complexes.

### Co-expression of BLIMP1 and IRF1 in human fetal intestine reveals a potential conserved functional role

Human placenta architecture, the duration of pregnancy, suckling behaviors and time frames of intestinal maturation have been adapted to fit the lifestyles of different mammalian species. In mice, crypt progenitors emerge from the inter-villous epithelium only after birth. Migration into the underlying mesenchyme leads to the formation of mature crypts and crypt-derived Blimp1-negative adult enterocytes gradually repopulate the intestinal epithelium during the suckling to weaning transition [16]. By contrast in humans overt cryptogenesis begins much earlier. Crypt-like structures initially appear in the small intestine *in utero* at approximately 12 weeks [45]. Considerable evidence suggests the gut epithelium retains its immature status throughout fetal development. Consistent with this human fetal enterocytes lack *ARG2* expression [46]. Additionally a human H4 fetal intestinal enterocyte cell line co-expresses *PRDM1* and very low levels of *ARG2* and *SIS* [47].

**Fig 7:**
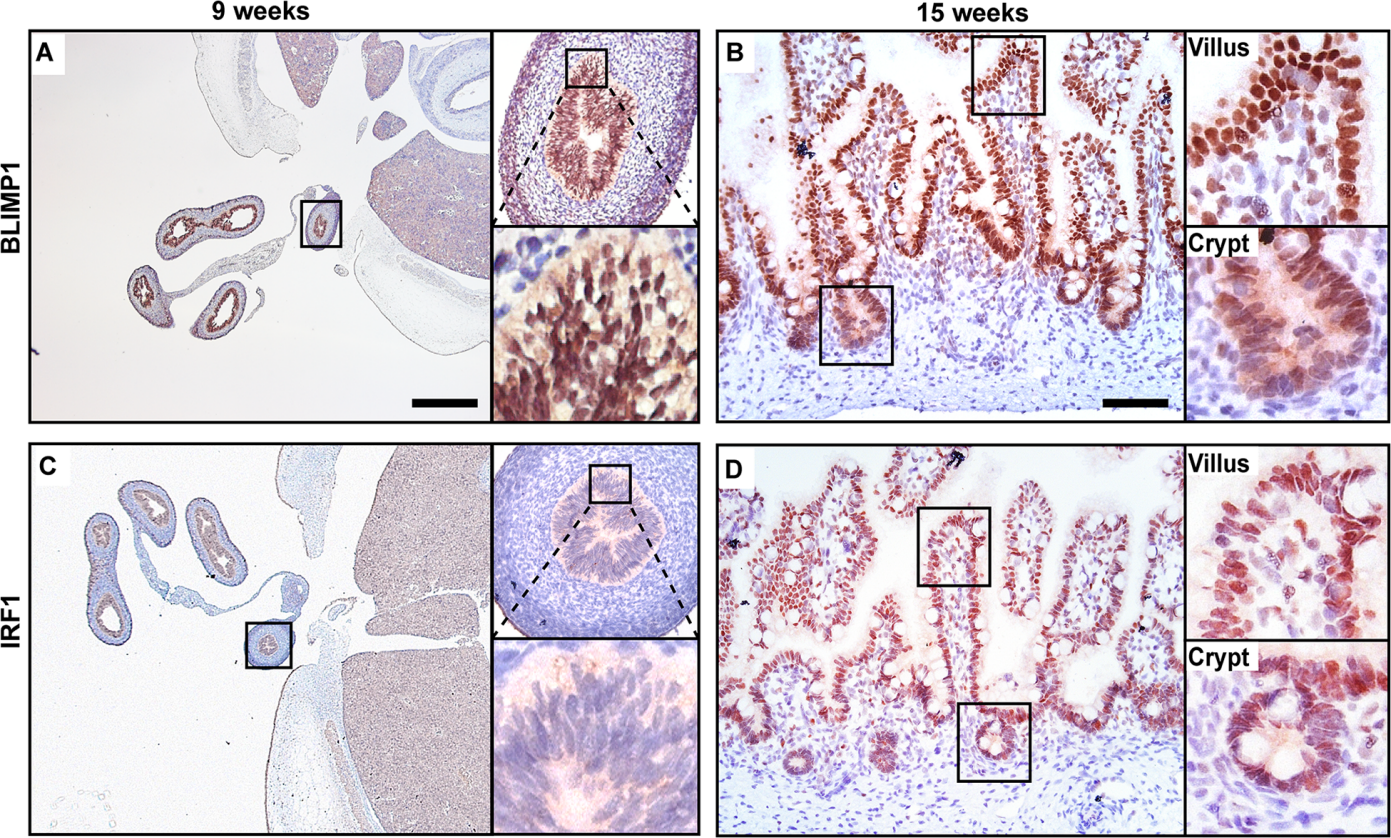
BLIMP1 and IRF1 are co-expressed in fetal human intestine. Nuclear BLIMP1 staining detected in the pseudostratified epithelium of the developing gut tube at (A) 9 weeks of gestation persists in (B) the villus epithelium including the immature crypts of Lieberkuhn at 15 weeks of gestation. (C) Although not detected in the gut tube prior to villus formation nuclear IRF1 staining is present in the villus epithelium at (D) 15 weeks of gestation. (A, C) Bar, 800 μm, (B, D) Bar 200 μm.

As shown in Fig 7A, immunohistochemistry demonstrate that BLIMP1 is constitutively expressed throughout the early epithelium at the pseudostratified stage and persists in the forming villous structures at 15 weeks of gestation (Fig 7B). In contrast IRF1 expression is undetectable in the gut epithelium prior to villus formation (Fig 7C). Slightly later at 15 weeks we detect IRF1 co-expressed together with BLIMP1 (Fig 7D). In humans BLIMP1 expression in crypt-like structures at late gestation pregnancy is probably required for continued production of immature enterocytes.

## Discussion

Target gene expression controlled by the zinc finger transcriptional repressor Blimp1/Prdm1 has been extensively characterized in the context of B-cell terminal differentiation to become antibody secreting plasma cells, the development of diverse CD4^+^ T lymphocyte subsets [4,48,49] as well as cell fate decisions governing effector versus regulatory CD8^+^ T cell homeostasis [12,50–53]. In guiding responses towards diverse antigenic stimuli, Blimp1 selectively shifts gene expression profiles in these lineage-committed precursors to directly influence developmental choices and ensure maximally effective protective immunity.

It has been known for many years that the Blimp1 consensus motif closely overlaps with the IRF-E sequence recognized by IRF1/IRF2 [20,29]. The structure of IRF1 bound to DNA reveals contacts mediated by a conserved cluster of tryptophan repeats [54]. In contrast the first two C2H2 zinc fingers of Blimp1 are required for recognition of its consensus motif [55]. Nonetheless, remarkably these structurally diverse proteins have the ability to interact with the same DNA sequence upstream of important target gene promoters.

The present experiments reveal considerable overlap between Blimp1 and Irf1 ChIP-Seq peaks. When further analyzed using Weeder, we identified an IRF-E DNA binding motif GAAAGTGAAA [20] underlying a subset of the 331 overlapping Blimp1/Irf1 ChIP-seq peaks. Of these 7% (n = 22) failed to match the motif, 23% (n = 75) contained a single match, and 70% (n = 234) contained 2 or more IRF-E sequences. For example, the overlapping peak proximal to the *Psmb10* promoter contained 8 motif matches. The *Tap1* and *Psmb8* bi-directional promoter also displays overlapping occupancy [56,57]. Closer inspection of the peak profiles reveals two closely adjacent Blimp1 peaks but only one overlaps with Irf1. The close arrangement of multiple adjacent consensus binding sites potentially allows co-occupancy of Blimp1 and Irf1 at these promoter regions. It is tempting to speculate that simultaneous binding of both repressor (Blimp1) and activator (Irf1) provides a rapid transcriptional switch mechanism.

Previous experiments demonstrate that MHC class I surface expression, absent at early embryonic stages of development is interferon-inducible [58,59]. Similarly temporal and spatially restricted Irf1 expression is tightly regulated in the early embryo [60]. *Irf1* constitutively expressed in the developing thymus plays an essential role in positive and negative selection of the CD8^+^ T cell repertoire [57,61]. MHC class I-restricted CD8^+^ T lymphocytes are directed against peptides derived from cytosolic proteins degraded by the proteasome that are translocated across the ER membrane by the TAP1/TAP2 transporter, stabilized by interactions with the dedicated class I chaperone Tapasin, and edited by ER aminopeptidases [19]. T cell receptor (TCR) repertoire selection is thought to depend on affinity differences as developing T cells interact with self-peptide MHC complexes displayed by thymic antigen-presenting cells in the cortical and medullary compartments [62]. During inflammatory and anti-viral responses the MHC class I peptide loading pathway becomes dramatically up-regulated to activate host defenses [31]. Interestingly both the adult thymus and small intestine constitutively express so-called immunoproteasome subunits [63]. We demonstrate here that numerous IFN-inducible components of the MHC class I antigen presenting pathway are direct Blimp1 targets in the embryonic intestinal epithelium. Irf1 constitutively expressed throughout intestinal development is poised to activate the MHC class I pathway in mature enterocytes coincident with down-regulated Blimp1 expression. In the neonatal intestine however, Blimp1 antagonizes Irf1 target gene expression to prevent premature activation of the MHC class I peptide-loading machinery.

Besides its essential role in absorption of nutrients, the intestinal epithelium also function as a protective barrier to prevent infection. The formation of gut-associated lymphoid tissues including the Peyer’s patches is initiated *in utero* but maturation of the mucosal immune system is incomplete at birth. Neonatal immune tolerance during postnatal colonization by commensal bacteria is essential for the establishment of a well-balanced host-commensal relationship. It is widely accepted that maternal tolerance of the allogenic fetus is largely dependent on the absence of embryonic MHC class I expression [58,59]. The present experiments demonstrate that another key feature of mammalian development is competitive binding by Blimp1 and Irf1 at the promoters of IFN-inducible components of the MHC class I machinery in fetal intestine to guarantee immune tolerance during postnatal intestinal maturation. We also identify BLIMP1 expression in human fetal enterocytes, implicating a potentially conserved functional role in guiding neonatal intestinal maturation and metabolic adaption. Although obtaining healthy preterm and neonatal material presents a considerable obstacle, nonetheless our future studies will aim to learn more about how BLIMP1 functions in humans to coordinate neonatal immune tolerance and the developmental switch from immature to mature enterocyte transcriptional programmes.

Note added in proof. While this manuscript was under review, another genome wide Blimp-1 ChIP data set was published by Saitou and colleagues (Kurimoto, K., Yabuta. Y., Hayashi, K. Ohta, H., Kiyonari, H., Mitani, T, Moritoki, Y., Kohri, K., Kimura, H., Yamamoto, T., Katou, Y, Shirahige, K., and M. Saitou (2015) Quantitative dynamics of chromatin remodelling during germ cell specification from mouse embryonic stem cells. Cell Stem Cell)[64]. The authors similarly used a Blimp-1-eGFP fusion knock-in strategy, but in contrast to our study, the eGFP cassette was inserted at the N-terminus and the resulting homozygous mice are fertile. They focused exclusively on expression during in vitro PGC specification, so it remains unclear whether germ cell defects in our BEG allele may reflect compromised recruitment of epigenetic partners and/or marginally reduced expression levels. Remarkably despite cell type differences, we see that there is roughly 48% overlap between the Blimp-1 ChIP seq peaks identified here in small intestine and those identified in PGCs by Kurimoto et al. Importantly overlapping targets include *Prdm1*, *Il33*, *Psmb9*, *Psmb10*, *Psmg4*, *Psme2*, *Tap1*, *Tap2*, *Tapbp*, and *Erap1*.

## Materials and Methods

### Generation of the Blimp1-eGFP knock-in allele

For the 5’ homology region, a StuI-XbaI fragment was excised from a genomic subclone [6] and introduced into a modified pBluescriptII plasmid (Stratagene). To complete the 5’ homology region PCR (Platinum Pfx polymerase, Invitrogen) was performed using the primers *Prdm1*-KI-F: AGAAACCAGCGCTTCTGTTTTAGTACGCGGAGC and *Prdm1*-KI-R: GAGAGGCGCGCCGAGAACTAGTCTCTGCCAGTCCTTGAAACTTCACGGAGCC with the bacterial artificial chromosome bMQ-375h16 as template. The PCR product was digested (XbaI, AscI) and cloned to introduce SpeI and AscI (underlined) cloning sites into *Prdm1* exon 3. The Blimp1-eGFP fusion construct plus SV40 polyadenylation signal [65] excised using XhoI-NotI was subcloned into a pBluscriptII based shuttle vector containing an upstream NheI site and downstream AscI site. This Blimp1-eGFP cDNA expression cassette was then ligated into SpeI and AscI sites in *Prdm1* exon 3. Finally, a fragment containing a Frt flanked Neomycin resistance cassette [66], the MfeI-SphI 3’ homology region [6] and Hsv-TK cassette were inserted using AscI and PmeI restriction sites. The NotI linearized targeting vector was introduced into CCE embryonic stem (ES) cells by electroporation.

Southern blot screening was performed as described using the restriction enzyme and probe combinations shown in Fig 1A [27]. Southern probes were amplified by PCR using the following primers: 5'-Forward: GATAGGATCCTTTCCAGCTGTTACTATGTAGG, 5'-Reverse: GATACTCGAGCTTATGCTTCATAGTTAATTTGG, 3'-Forward: GATAGAATTCAATGCCATTTGTCAGGGAGC, 3'-Reverse: GATACTCGAGCTTTTGGCCACAGGACAATG. For excision of the Frt-Neo cassette, correctly targeted ES cell clones were transiently transfected with pCAGGS-FlpO (generously provided by Bill Skarnes) and subsequently screened using the 3' probe together with KpnI genomic digest.

### Animals

Genotyping of mice carrying the novel eGFP knock-in (BEG) allele was performed with Forward primers BEG Mut (GTTATTGGCGTGGTAAGTAAGG) and WT (AGGCATCCTTACCAAGGAAC) and Reverse primers BEG Mut (ATTTATCACTGTGAGCTCTCCAG) and WT (GCTGAAGGGAGGAAGAAATG). The cycling conditions were 94°C for 20 s, 58°C for 30 s, and 72°C for 45 s for 35 cycles. Conditional mutants selectively lacking Blimp1 function in the developing intestine, generated by crossing *Prdm1* ^CA/CA^ animals to the Villin cre transgenic strain were genotyped as described [16]. The targeted deletion that selectively eliminates exon 1A promoter usage and disrupts Blimp1 dependent plasma cell differentiation as well as the BAC transgenic reporter strain expressing membrane targeted Venus under the control of the Blimp1 regulatory elements have been described [25,27]. All animal experiments were performed in accordance with Home Office (UK) regulations and were approved by the University of Oxford Local Ethical Committee.

### Western blot analysis

Cell cultures to induce plasma cell differentiation and Western blot analysis of LPS-stimulated splenocytes were performed as described previously [65]. To insure quantitative protein recoveries, embryonic and postnatal intestinal tissues were flushed with PBS, snap frozen and immediately dissociated directly in SDS sample buffer. The primary antibodies were: rat monoclonal anti-Blimp1 (clone 5E7, SC-130917; Santa Cruz), rabbit polyclonal anti-Irf1 rabbit (M-20, Santa Cruz, SC-640), rat monoclonal anti-MHC class I (ER-HR52, SC-59199; Santa Cruz), hamster monoclonal anti-G9a (clone 14–1, D141-3; MBL), rabbit polyclonal anti-HDAC1 (AB7028; Abcam), rabbit polyclonal anti-HDAC2 (AB7029; Abcam), rabbit monoclonal anti-LSD1 (clone EPR6825, AB129195; Abcam), rabbit polyclonal anti-CoREST (07–455, Merck Millipore) and rabbit polyclonal anti-β-tubulin (SC-9104; Santa Cruz). Secondary antibodies were anti-mouse immunoglobulin (Ig)–horseradish peroxidase (HRP) (NA931V; GE Healthcare), anti-rat Ig—HRP (NA935V; GE Healthcare), anti-rabbit Ig—HRP (NA934V; GE Healthcare), or anti-Armenian hamster Ig—HRP (SC-2904; Santa Cruz). Levels of Blimp1-eGFP fusion protein relative to levels of endogenous Blimp1 protein were quantified on Western blots using a ChemiDoc XRS+ system and Image Lab software (BIO-RAD).

### Immunofluorescence analysis

Cell staining experiments were performed as described [65] using a FACSCalibur flow cytometer (BD Biosciences), and data were analyzed with FlowJo software (Tree Star). High-resolution images were captured using an ImageStreamX Mk II imaging flow cytometer (AMNIS) and analyzed using IDEAS software (AMNIS).

### Histology and immunohistochemistry

Placental and intestinal tissue samples were fixed overnight in 4% paraformaldehyde (PFA) in PBS, dehydrated in ethanol, embedded in paraffin and sectioned (6 μm) Immunohistochemistry was performed as described [8]. Primary antibodies were rat monoclonal anti-Blimp1 (clone 5E7, SC-130917; Santa Cruz), or for human tissue rat monoclonal anti-Blimp1 (clone 6D3, 14-5963-82; eBioscience), rabbit monoclonal anti-Irf1 (8478; Cell signaling) or rat monoclonal anti-MHC class I (ER-HR52, SC-59199; Santa Cruz). Visualization of primordial germ cells, by staining for alkaline phosphatase was performed as described [67]. Testes samples were fixed overnight in Bouin’s fixative, dehydrated in ethanol, embedded in paraffin, sectioned (6 μm) and stained with hematoxylin and eosin.

### ChIP-seq analysis

Small intestines were dissected, flushed with PBS, cut into small pieces and cross-linked with 1% formaldehyde in PBS for 15 min at 4°C followed by 35 min at 25°C [68] and subsequently processed for ChIP using either 10 μg of mouse anti-GFP IgG2a (clone 3E6, A11120; Invitrogen), polyclonal rabbit anti-Irf1 antibody (M-20, Santa Cruz, SC-640) or as a control, normal rabbit IgG (Santa Cruz, SC-2027) as described previously [28]. The DNA samples were multiplexed and sequenced using two lanes on an Illumina HiSeq 2000 sequencer. Duplicate test (GFP ChIP of *Prdm1* ^BEG/BEG^, or Irf1 ChIP of WT and Villin-cre conditional Blimp1 mutants, [16]) samples or individual negative control (GFP ChIP of wild type or, normal rabbit IgG ChIP) and input samples were analyzed.

Sequence reads were mapped to the mm9 mouse genome release with Stampy using default parameters [69]. Peak calling was performed with MACS1.4.2 [70,71], using default parameters to call areas of enrichment in ChIP samples over input. Regions of enrichment detected in negative controls samples were removed from subsequent analysis. GFP ChIP peaks called in wild type samples were subtracted from GFP peaks called in BEG/BEG samples. Similarly, normal rabbit IgG ChIP peaks were subtracted from Irf1 peaks. The overlapping peaks in duplicate ChIP samples were then identified and the core region of overlap was further analyzed. The genomic distribution of ChIP-seq peaks compared to gene annotations was determined using CEAS [72]. Genes of Ensembl release 67 with proximal Blimp1 or Irf1 binding were identified using custom Perl scripts. *De novo* identification of motifs within ChIP-seq peaks was performed using MEME suite tools [73]. Functional annotation of ChIP-seq peaks was performed with GREAT version 2.0.2 using the basal plus extension rule, annotating genes within 5 kb of transcription start sites initially or within 25 kb when no proximal gene in known to exist [74]. Regions of overlap between the ChIP-seq peaks identified in present study with other published datasets were compared using custom Perl scripts. Peak regions in human datasets were first converted to mm9 using the UCSC liftOver function. The association between Blimp1 binding (± 5kb of TSS) and genes differentially expressed in embryonic *Prdm1* ^-/-^ small intestine [16] (Gene Expression Omnibus database, www.ncbi.nlm.nih.gov/geo, accession no. GSE29658) was calculated by chi-square test.

For comparative Irf1 ChIP-seq analysis reads mapping within identified peaks were counted using HTSeq-count [75]. TMM normalization factors and tagwise dispersions were then computed, and differential occupancy between sites in mutant and wild type determined by exact test using edgeR [76,77]. False discovery rates were calculated using the Benjamini and Hochberg method. Sites differentially bound with an FDR ≤ 0.05 were considered significant.

### qPCR validation

QPCR analysis of triplicate GFP or control IgG ChIP, and input samples of *Prdm1* ^*BEG/BEG*^ and WT E18.5 intestine was performed using QuantiTech SYBR Green master mix (Q2040143; Qiagen) on a Rotor-Gene Q (Qiagen). Primers were designed to amplify 100–200 bp regions central to ChIP-seq peak genomic coordinates. Selected genes included *c-Myc*, *Psmb8*, *Psmb9*, *Tap1*, *Tap2 and Tapbp* [20,24] and several candidates with increased transcript levels in Blimp1 mutants i.e. *Prdm1* (45.3 fold), *Slc16a5* (16.1 fold), *Gsta1* (16.8 fold), *2210407C18Rik* (15.7 fold), *Trib3* (2.9 fold) and *Cyp4v3* (68.9 fold) [16]. A non-enriched ChIP-seq region in the 3’UTR of the *Prdm1* gene was used as a negative control. Primer sequences are shown in S6 Dataset. Fold enrichment of ChIP over input was determined relative to a standard curve generated from log diluted sheared genomic DNA.

### RT-PCR

Total RNA from yolk sac and embryonic small intestinal tissues was isolated using an RNeasy Mini kit (Qiagen), and reverse transcription-PCR (RT-PCR) was performed using the OneStep RT-PCR kit (Qiagen) as previously described [27]. Primers Ex1AFor and Ex1BFor in combination with Ex3Rev distinguish *Prdm1* exon 1A and *Prdm1* exon 1B transcripts whereas total *Prdm1* transcripts were detected with primers Ex4For and Ex5Rev [27].

### Accession numbers

The ChIP-seq data have been deposited in NCBI GEO with the accession number GSE66069.

## Supporting Information

S1 Fig Q-PCR validation of Blimp1 occupancy. Real-time PCR quantification of triplicate E18.5 *Prdm1* ^BEG/BEG^ small intestine GFP ChIP samples relative to input DNA. GFP ChIP of wild type (WT) and normal mouse IgG ChIP of *Prdm1* ^BEG/BEG^ and wild type were included as negative controls. A region within the 3’UTR of *Prdm1* was used as an internal control. Significant enrichment of all genomic regions corresponding to Blimp1 ChIP-seq peaks was observed selectively in GFP-ChIP of *Prdm1* ^BEG/BEG^ samples. Data represent mean % of input +/- SEM of triplicate samples per group. *P<0.05 relative to WT GFP ChIP samples.
(PDF)

S2 Fig RT-PCR analysis demonstrates alternative *Prdm1* promoter usage in yolk sac and embryonic small intestine. Primers Ex1AFor and Ex1BFor in combination with Ex3Rev1 distinguish *Prdm1* exon 1A and *Prdm1* exon 1B transcripts whereas total *Prdm1* transcripts were detected with Primers Ex4For and Ex5Rev. A STO fibroblast sample is included as a negative control.
(PDF)

S3 Fig Blimp1 ChIP-seq peaks contain known BLIMP1 and IRF-1 binding motifs. *De novo* analysis (MEME) of all Blimp1 ChIP-seq peaks (n = 2689) identified a binding motif with significant similarity to both BLIMP1 and IRF1 motifs (JASPAR_ CORE_2014_Vertebrates database).
(PDF)

S4 Fig Irf1 preferentially binds proximal to TSS. (A) Distribution of all Irf1 peak locations in comparison to whole genome at defined genomic regions. * *P* = 1.8 x 10^−119^ in comparison to whole genome for the same region. (B) The distance of each Irf1 ChIP-seq peak from the nearest TSS binned at 5kb intervals.
(PDF)

S5 Fig Western blot analysis of Blimp1/*Prdm1* chromatin-modifying corepressors. Lsd1, CoREST, Hdac1/2 and G9a expression levels in embryonic and postnatal small intestine. We observed roughly similar Lsd1, CoREST, Hdac1/2 and G9a expression levels at embryonic (E16.5) through to post weaning stages (P28). A STO fibroblast sample is included as a negative control for Blimp1 expression.
(PDF)

S1 Dataset Annotation of Blimp1 peaks identified by ChIP-seq. (XLSX)

S2 Dataset Overlapping Blimp-1 peaks between our dataset and those identified in HeLa S3 and transfected P19 EC cells. (XLSX)

S3 Dataset Genes altered in Blimp-1 mutants (*P*≤ 0.05, Harper *et al*., 2011) with Blimp-1 binding ±25 kb TSS. (XLSX)

S4 Dataset Annotation of Irf1 peaks and those shared with a LPS-treated BMDC Irf1 ChiP-seq dataset. (XLSX)

S5 Dataset Overlapping Blimp1/Irf1 peaks and the number of predicted binding sites within peak regions. (XLSX)

S6 Dataset Q-PCR primers used in this study. (XLSX)
