# Supplementary figures and images for: Blimp1/Prdm1 Functions in Opposition to Irf1 to Maintain Neonatal Tolerance during Postnatal Intestinal Maturation
Source: PLoS Genet. 2015 Jul 9;11(7):e1005375. doi: 10.1371/journal.pgen.1005375 (PMC4497732; doi:10.1371/journal.pgen.1005375)

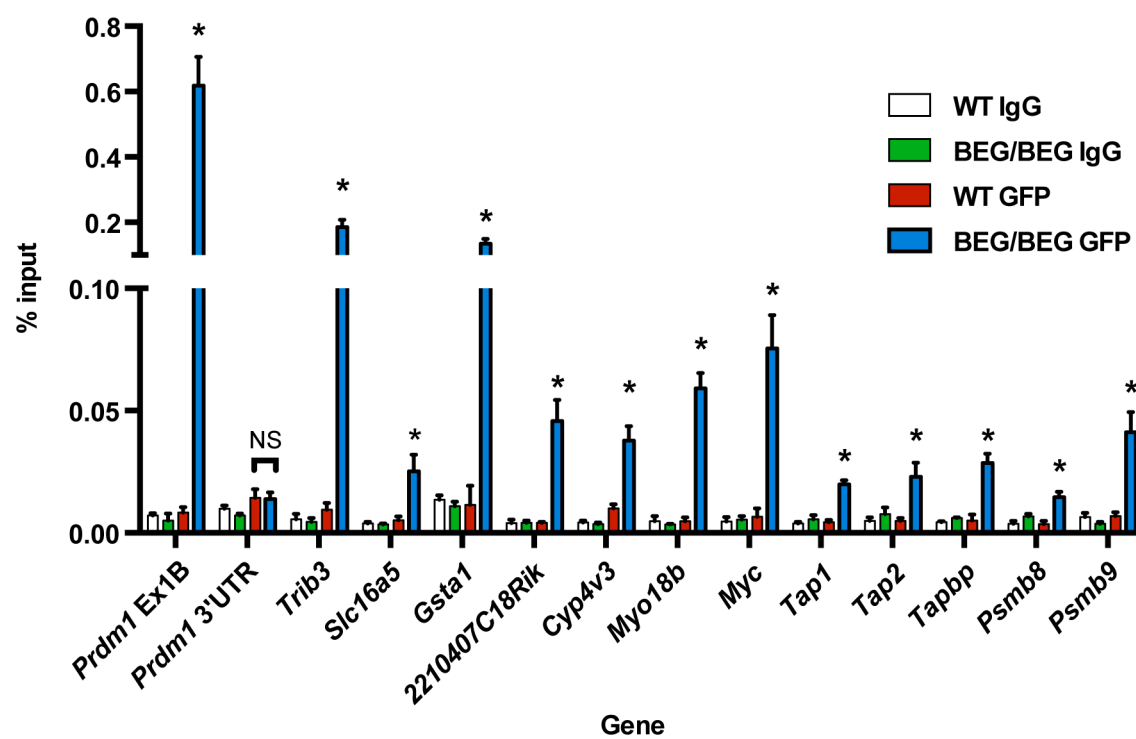

Supplement: S1 Fig — Real-time PCR quantification of triplicate E18.5 Prdm1 BEG/BEG small intestine GFP ChIP samples relative to input DNA. GFP ChIP of wild type (WT) and normal mouse IgG ChIP of Prdm1 BEG/BEG and wild type were included as negative controls. A region within the 3’UTR of Prdm1 was used as an internal control. Significant enrichment of all genomic regions corresponding to Blimp1 ChIP-seq peaks was observed selectively in GFP-ChIP of Prdm1 BEG/BEG samples. Data represent mean % of input +/- SEM of triplicate samples per group. *P<0.05 relative to WT GFP ChIP samples. (PDF) [file pgen.1005375.s001.pdf]

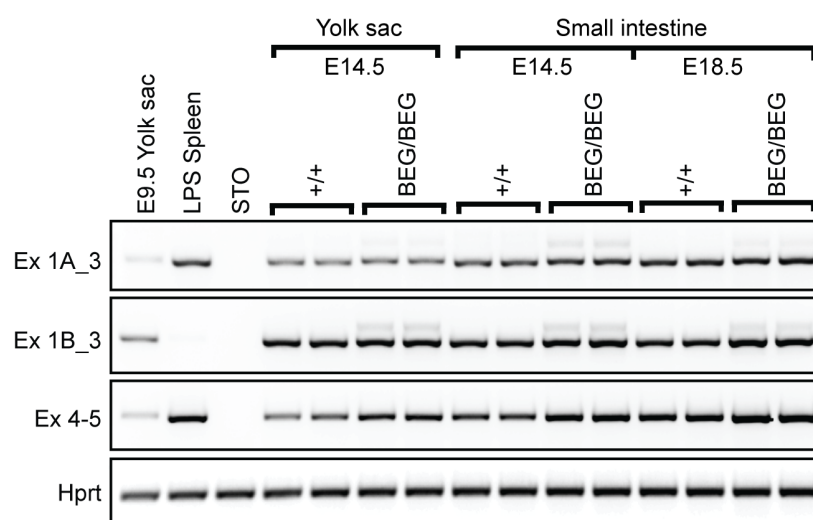

Supplement: S2 Fig — Primers Ex1AFor and Ex1BFor in combination with Ex3Rev1 distinguish Prdm1 exon 1A and Prdm1 exon 1B transcripts whereas total Prdm1 transcripts were detected with Primers Ex4For and Ex5Rev. A STO fibroblast sample is included as a negative control. (PDF) [file pgen.1005375.s002.pdf]

**A**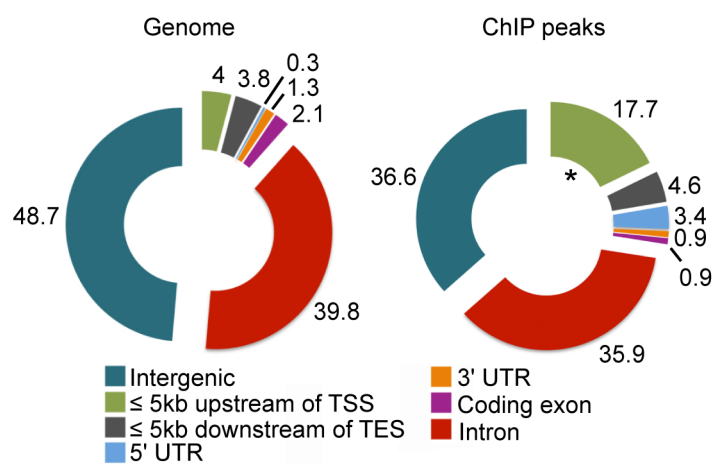**B**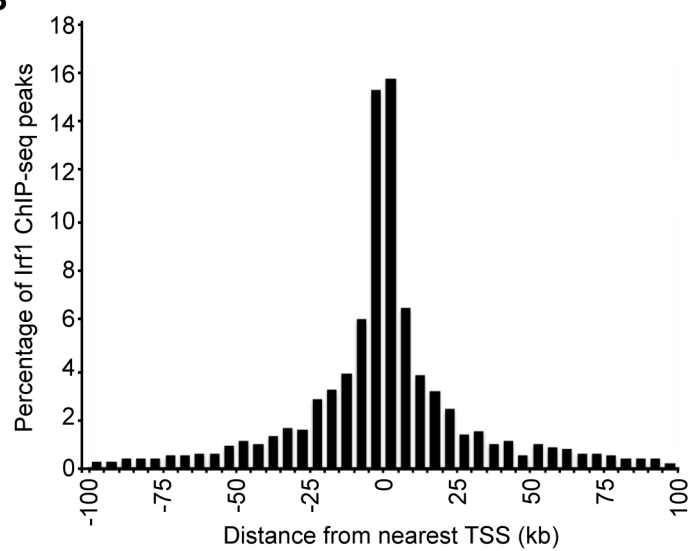

Supplement: S4 Fig — (A) Distribution of all Irf1 peak locations in comparison to whole genome at defined genomic regions. * P = 1.8 x 10−119 in comparison to whole genome for the same region. (B) The distance of each Irf1 ChIP-seq peak from the nearest TSS binned at 5kb intervals. (PDF) [file pgen.1005375.s004.pdf]

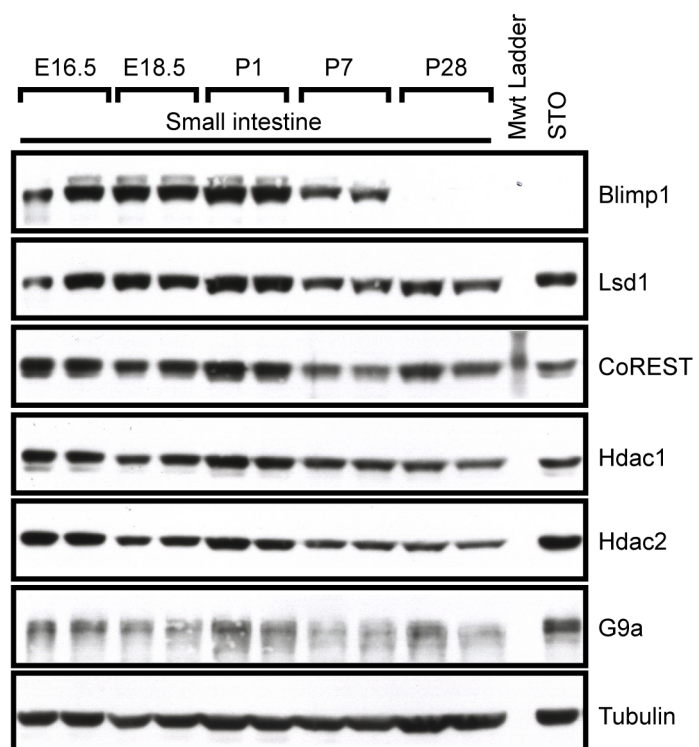

Supplement: S5 Fig — Lsd1, CoREST, Hdac1/2 and G9a expression levels in embryonic and postnatal small intestine. We observed roughly similar Lsd1, CoREST, Hdac1/2 and G9a expression levels at embryonic (E16.5) through to post weaning stages (P28). A STO fibroblast sample is included as a negative control for Blimp1 expression. (PDF) [file pgen.1005375.s005.pdf]
