# Supplementary material for: Blimp1/Prdm1 Functions in Opposition to Irf1 to Maintain Neonatal Tolerance during Postnatal Intestinal Maturation
Source: PLoS Genet. 2015 Jul 9;11(7):e1005375. doi: 10.1371/journal.pgen.1005375 (PMC4497732; doi:10.1371/journal.pgen.1005375)

Name	MA0508.1
Database	JASPAR_CORE_2014_vertebrates.meme
Alt. Name	BLIMP1/PRDM1
P value	2.8183 x10^{-9}
E value	1.6656 x10^{-6}
q value	3.3310 x10^{-6}
Overlap	14
Offset	1
Orientation	Normal

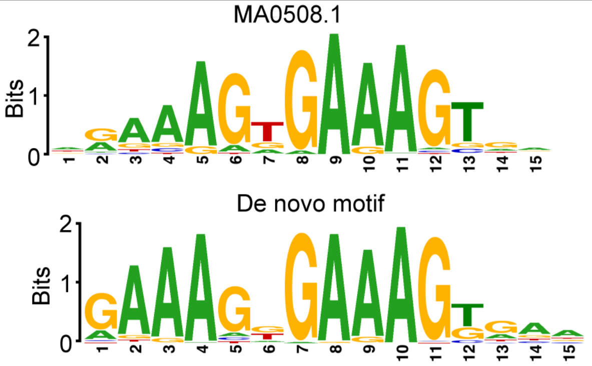

Name	MA0050.2
Alt. Name	IRF1
Database	JASPAR_CORE_2014_vertebrates.meme
P value	2.8366 x10^{-8}
E value	1.6765 x10^{-5}
q value	1.6763 x10^{-5}
Overlap	15
Offset	5
Orientation	Reverse Complement

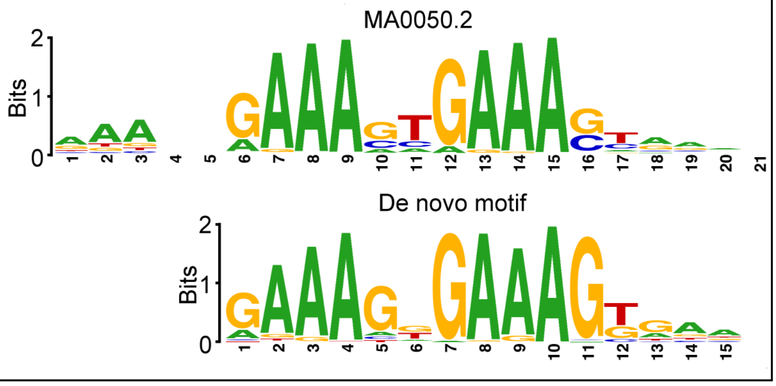

Supplement: S3 Fig — De novo analysis (MEME) of all Blimp1 ChIP-seq peaks (n = 2689) identified a binding motif with significant similarity to both BLIMP1 and IRF1 motifs (JASPAR_ CORE_2014_Vertebrates database). (PDF) [file pgen.1005375.s003.pdf]
